# Effect of Intranasal vs Intramuscular Naloxone on Opioid Overdose: A Randomized Clinical Trial

**DOI:** 10.1001/jamanetworkopen.2019.14977

**Published:** 2019-11-13

**Authors:** Paul Dietze, Marianne Jauncey, Allison Salmon, Mohammadreza Mohebbi, Julie Latimer, Ingrid van Beek, Colette McGrath, Debra Kerr

**Affiliations:** 1 Behaviours and Health Risks Program, Burnet Institute, Melbourne, Victoria, Australia; 2 School of Public Health and Preventive Medicine, Monash University, Clayton, Victoria, Australia; 3 Uniting Medically Supervised Injecting Centre, Kings Cross, New South Wales, Australia; 4 Biostatistics Unit, Faculty of Health, Deakin University, Burwood, Victoria, Australia; 5 South Eastern Sydney Local Health District, New South Wales, Australia; 6 Kirby Institute, University of New South Wales, Sydney, Sydney, New South Wales, Australia; 7 Justice Health Forensic Mental Health Network, New South Wales Health, Randwick, New South Wales, Australia; 8 Centre for Quality and Patient Safety, School of Nursing and Midwifery, Deakin University, Geelong, Australia

## Abstract

**Importance:**

Previous unblinded clinical trials suggested that the intranasal route of naloxone hydrochloride was inferior to the widely used intramuscular route for the reversal of opioid overdose.

**Objective:**

To test whether a dose of naloxone administered intranasally is as effective as the same dose of intramuscularly administered naloxone in reversing opioid overdose.

**Design, Setting, and Participants:**

A double-blind, double-dummy randomized clinical trial was conducted at the Uniting Medically Supervised Injecting Centre in Sydney, Australia. Clients of the center were recruited to participate from February 1, 2012, to January 3, 2017. Eligible clients were aged 18 years or older with a history of injecting drug use (n = 197). Intention-to-treat analysis was performed for all participants who received both intranasal and intramuscular modes of treatment (active or placebo).

**Interventions:**

Clients were randomized to receive 1 of 2 treatments: (1) intranasal administration of naloxone hydrochloride 800 μg per 1 mL and intramuscular administration of placebo 1 mL or (2) intramuscular administration of naloxone hydrochloride 800 μg per 1 mL and intranasal administration of placebo 1 mL.

**Main Outcomes and Measures:**

The primary outcome measure was the need for a rescue dose of intramuscular naloxone hydrochloride (800 μg) 10 minutes after the initial treatment. Secondary outcome measures included time to adequate respiratory rate greater than or equal to 10 breaths per minute and time to Glasgow Coma Scale score greater than or equal to 13.

**Results:**

A total of 197 clients (173 [87.8%] male; mean [SD] age, 34.0 [7.82] years) completed the trial, of whom 93 (47.2%) were randomized to intramuscular naloxone dose and 104 (52.8%) to intranasal naloxone dose. Clients randomized to intramuscular naloxone administration were less likely to require a rescue dose of naloxone compared with clients randomized to intranasal naloxone administration (8 [8.6%] vs 24 [23.1%]; odds ratio, 0.35; 95% CI, 0.15-0.66; *P* = .002). A 65% increase in hazard (hazard ratio, 1.65; 95% CI, 1.21-2.25; *P* = .002) for time to respiratory rate of at least 10 and an 81% increase in hazard (hazard ratio, 1.81; 95% CI, 1.28-2.56; *P* = .001) for time to Glasgow Coma Scale score of at least 13 were observed for the group receiving intranasal naloxone compared with the group receiving intramuscular naloxone. No major adverse events were reported for either group.

**Conclusions and Relevance:**

This trial showed that intranasally administered naloxone in a supervised injecting facility can reverse opioid overdose but not as efficiently as intramuscularly administered naloxone can, findings that largely replicate those of previous unblinded clinical trials. These results suggest that determining the optimal dose and concentration of intranasal naloxone to respond to opioid overdose in real-world conditions is an international priority.

**Trial Registration:**

anzctr.org.au Identifier: ACTRN12611000852954.

## Key Points

**Question:**

Is 800 μg of naloxone hydrochloride administered intranasally as effective in reversing opioid overdose as the same dose administered intramuscularly?

**Findings:**

In this double-blind, double-dummy randomized clinical trial of 197 clients in a medically supervised injecting facility, significantly more clients who received naloxone intranasally required a rescue dose of naloxone compared with clients given naloxone intramuscularly, reflecting a slower time to respond in terms of improved respiration and consciousness among the intranasal group.

**Meaning:**

This trial found that the same dose of naloxone given intranasally was not as effective as naloxone given intramuscularly in reversing opioid overdose, suggesting that further work is needed to establish the optimal dose of nasal naloxone.

## Teaser

This randomized clinical trial compares 2 administration methods for the opioid antagonist naloxone in individuals served by a medically supervised drug-injecting facility in Australia.

## Introduction

Naloxone hydrochloride is a highly effective opioid antagonist that has been used in medical practice to reverse the effects of opioid use for more than 40 years.^1^ Its use is particularly important in cases of opioid overdose, and it is listed as an essential medicine by the World Health Organization. Intramuscular or intravenous injection of naloxone is common for overdose reversal, but it can also be effective when administered intranasally.^2,3,4,5^ However, few randomized trials have examined the efficacy of alternate naloxone administration routes. Nevertheless, take-home naloxone programs in the United States and elsewhere have been offering devices that permit intranasal administration in tandem with the naloxone designed for intramuscular administration.^6,7,8^

The intranasal route for naloxone administration holds promise, with naloxone absorption possible through the nasal mucosa.^9^ Published pharmacokinetic data initially suggested that the intranasal route was inefficient compared with the widely used intramuscular route,^10^ but recent work with more concentrated forms suggests the intranasal route has slower onset of action but adequate bioavailability after 5 to 20 minutes with a range of naloxone doses.^11,12,13,14,15^ These findings are consistent with the results of 2 trials of overdose reversal conducted with paramedics in the out-of-hospital setting,^3,5^ with slightly slower overdose reversal times and an increased need for intramuscular rescue doses observed in the intranasal groups in both trials. Both studies compared 2 mg of naloxone hydrochloride, but 1 trial used a more concentrated preparation (2 mg per 5 mL^3^ solution vs 2 mg per 1 mL^5^ solution); roughly comparable results were found despite the likely waste of dose with the weaker concentration. However, neither of these trials allowed for blinding of the paramedics, which may have introduced treatment bias that, in turn, may explain the difference in the treatment effects,^16^ including increased propensity to use rescue doses in the intranasal groups.^5^

In this prospective, double-blind, double dummy randomized clinical trial, we examined whether a dose of intranasal naloxone hydrochloride 800 μg per 1 mL solution is as effective as the same dose of intramuscular naloxone for the reversal of acute opioid overdose. On the basis of previous trial results, we hypothesized that the efficacy of intranasal naloxone would match that of intramuscular naloxone in terms of the need for an additional rescue dose of intramuscular naloxone, given that previous unblinded trials were subject to bias.

## Methods

This double-blind, double-dummy randomized clinical trial was approved by the ethics committee of the South East Sydney Local Health District. Verbal informed consent was provided by all participants. Clinical Trial Notification of the use in this trial of an unapproved medical product was recorded with the Australian Therapeutic Goods Administration. An independent data and safety monitoring board provided oversight of the trial, and monthly reports were provided by the study coordinator (A.S.). This study followed the Consolidated Standards of Reporting Trials (CONSORT) reporting guideline.^17^ See Supplement 1 for the trial protocol.

### Design and Participants

**Figure 1.**
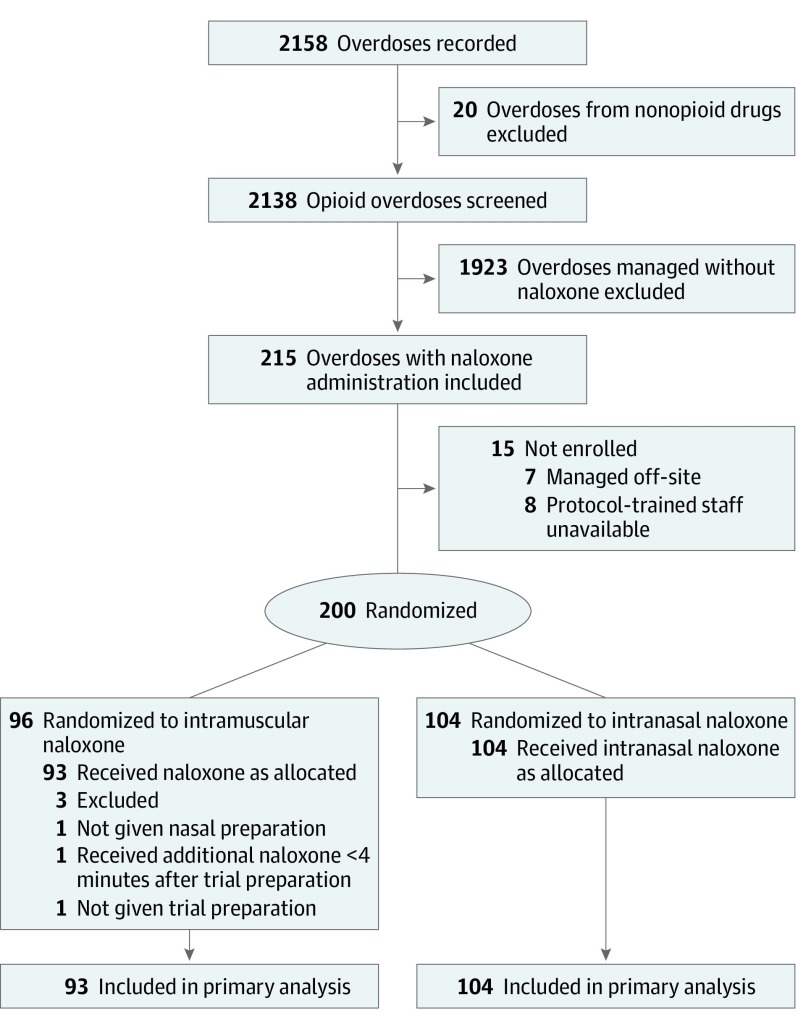
CONSORT Diagram of Participant Flow

In this trial, participants were randomized 1:1 to receive 800 μg of naloxone hydrochloride per 1 mL solution, either intranasally or intramuscularly (Figure 1). All participants received an intramuscular injection (active or placebo) and an intranasal spray (active or placebo). We tested for differences between the 2 routes of administration, powered by the differences observed in the primary outcome of the need for a rescue dose of naloxone in previous research.^3,5^

Participants were recruited consecutively among clients of the Uniting Medically Supervised Injecting Centre (MSIC) in Sydney, Australia, from February 1, 2012, to January 3, 2017. The MSIC serves a particularly vulnerable group of clients who typically have a long history of social and economic deprivation. The MSIC allows eligible clients (those aged 18 years or older with a history of injecting drug use) to inject preobtained drugs under the supervision of clinically trained staff. Clients are observed postinjection for signs of overdose. Internal management protocols are part of the legislative basis for the service, which means that clients already intoxicated from alcohol or other drugs or those accompanied by a child are excluded from entry into the facility. All MSIC clients are required to register before first use of the service. For each subsequent service occasion, clients self-report which drug or drugs they intend to inject for that visit.

As potential participants in this trial, all registered clients were informed of the study through extensive advertising within the MSIC. Clients were asked verbally if they were willing to consent to participate, and declines were documented in the administrative database. All consenting clients with symptoms or signs of an opioid overdose that required naloxone administration were eligible for entry on the basis of existing and approved MSIC protocols and clinical criteria for overdose, which included reduced level of consciousness as measured by the Glasgow Coma Scale score (GCS score of <13; score range: 3-15, with the highest score indicating normal responses), respiratory depression (respiratory rate [RR] of <10 breaths per minute), or reduced oxygen saturations (<95%) as measured by pulse oximetry. Clients could be enrolled in the study multiple times if they experienced an overdose on more than 1 occasion and on each occasion would be randomized according to the trial method. Consenting clients were informed of their participation after the intervention and fully regaining consciousness. The MSIC had 249 607 presentations with intention to inject by approximately 730 individuals per month across the 47-month study period. The staff managed 2158 overdose events, of which 215 (10.0%) involved the naloxone protocol.

Naloxone and placebo were manufactured and prepared in 2 batches (first by Sypharma Pty Ltd and then by GD Pharma). Labels and randomization sequence were prepared by a packager (Pharmpackpro Pty Ltd) and sent to the manufacturers. The switch between manufacturers occurred because the initial batch of naloxone drug packs expired and the availability of additional product was delayed, which led to the trial being suspended from August 7, 2014, to December 8, 2015.

### Randomization, Masking, and Study Treatment

A computer-generated 1:1 randomization schedule along with primary labeling for placebo and active naloxone study packs were provided by the packager. Each study pack contained 2 vials, 1 of which was labeled intramuscular and the other intranasal; 1 contained active naloxone, and the other contained placebo solution (water from Sypharma and saline from GD Pharma). The packs were sequentially numbered, coded according to the treatment arm, and allocated sequentially to participants in strict order of their registration for the trial. All vials for use in the study were identical in design and labeling apart from study codes. No direct contact at any stage occurred between the generator and executors of the assignment, which meant the participants, the nurses administering the naloxone, and the researchers were all blinded to the 2 treatment arms. Treatment allocations were decoded after statistical analysis.

Details of treatment methods are described in detail in the study protocol. Briefly, MSIC staff managed drug overdoses using existing clinical protocols. These protocols state that a client would receive airway management and oxygenation either via a mask or artificial ventilation (bagging) for 5 minutes and then would be assessed for the need for naloxone. If a consenting client’s response after 5 minutes was inadequate (oxygen saturation not maintained at ≥95% or with GCS score <13 or RR <10), the client was enrolled into the study, and trial drugs were administered by a registered nurse in accordance with existing standing orders from the MSIC medical director (M.J.).

Participants were randomized to receive naloxone in 1 of 2 forms: (1) intranasal administration of naloxone hydrochloride 800 μg per 1 mL and intramuscular administration of placebo 1 mL, or (2) intramuscular administration of naloxone hydrochloride 800 μg per 1 mL and intranasal administration of placebo 1 mL. The 800-μg intramuscular dose has been used throughout the operation of the MSIC and is consistent with paramedic clinical practice guidelines in New South Wales,^18^ but the dose is higher than the 400 μg dose recommended by the World Health Organization.^19^ Two MSIC staff members attended the patient during treatment of opioid overdose, and a third attending staff member recorded study information on a data collection form.

For intranasal administration, the contents of the vial were drawn into 1 syringe, and the syringe was attached to a mucosal atomization device. Each nostril received 0.5 mL (400 μg), with rapid depression of the syringe to achieve adequate atomization. For intramuscular administration, following the standard practice, the full 1-mL dose was drawn into a single 3-mL syringe and injected into the deltoid muscle with a 23-gauge needle. The order of drug administration was altered approximately midway through the study. Initially, the intranasal route was first, followed by the intramuscular route, but the order was reversed for the second half of the study.

Supportive care or oxygenation was administered simultaneously in accordance with existing MSIC clinical protocols. Any client who failed to respond adequately (ie, GCS score remained <13, RR remained <10, or oxygen saturation remained <95%) after 10 minutes was given a second rescue dose of 800 μg of intramuscular naloxone hydrochloride. This dose was not subject to randomization.

### Outcome Measures, Sample Size, and Statistical Power

Outcome measures were assessed at baseline and at 10 minutes after treatment. The primary outcome measure was the requirement for a secondary dose of naloxone at 10 minutes. Secondary outcome measures included response time that was indexed in 2 ways: (1) time to effective and spontaneous respirations at a rate of greater than or equal to 10 per minute, and (2) time to GCS score greater than or equal to 13. These criteria have been used in previous research in this field.^5^

An adverse event was defined as any untoward medical occurrence in a participant that may or may not have a causal relationship with the study treatment. An adverse event can, therefore, be any unfavorable or unintended sign, symptom, condition, and/or observation that may or may not be associated with the study treatment. A serious adverse event was defined as any untoward medical occurrence that resulted in death; persistent or substantial disability or incapacity; or a condition requiring medical or surgical intervention and/or hospitalization, such as cardiac arrest, acute pulmonary edema, cardiac arrhythmias, or epileptic seizure.

Previous research in this field found a 14% difference in incidence in the primary outcome measure.^5^ The planned sample size was 99 participants in each group, which, at a 2-sided 5% significance level, provided more than 80% power to detect a difference of 14% in the requirement for secondary naloxone in the comparison of intranasal with intramuscular naloxone administration.

### Statistical Analysis

Descriptive analyses were conducted to compare intranasal and intramuscular groups for observed differences in demographic data and potential confounding variables shown in Table 1 and Table 2. These analyses were conducted in accordance with the International Conference on Harmonization E9 statistical principles.^20^ An intention-to-treat analysis was performed for all participants who received both intranasal and intramuscular modes of treatment. The main analysis for comparison of incidence rate of secondary naloxone was performed using generalized linear mixed effects modeling for binary outcome variables with a logistic link function to account for within-patient clustering.^21^ A generalized estimating equation approach with an unstructured covariance pattern was implemented to account for within-client clustering (autocorrelation).^21^ Odds ratios (ORs) and 95% CIs were reported as treatment effect.

**Table 1.** Event Characteristics

Variable	No. (%)	
	Intramuscular Administration (n = 93)	Intranasal Administration (n = 104)
Sex		
Male	81 (87.1)	92 (88.5)
Female	12 (12.9)	12 (11.5)
First language		
English	85 (91.4)	97 (93.3)
Other	8 (8.6)	7 (6.7)
Country of birth		
Australia	86 (92.5)	101 (97.1)
Other	7 (9.7)	3 (2.9)
Aboriginal or Torres Strait Islander status^a^		
Yes	14 (15.4)	13 (13.0)
No	77 (84.6)	87 (87.0)
Any blood-borne virus^a^		
Yes	46 (50.5)	50 (49.0)
No	45 (49.5)	52 (51.0)
Individual clients	60 (64.5)	67 (64.4)
Age, mean (SD) [range], y	33.6 (7.5) [19-56]	34.4 (8.1) [20-55]
First injecting age, mean (SD) [range], y	19.6 (6.7) [11-38]	19.6 (7.8) [11-54]

^a^ Some data were missing, so the sample size was less than 197.

**Table 2.** Event-Level Overdose Characteristics and Attributions by Treatment Group

Variable	No. (%)	
	Intramuscular Administration (n = 93)	Intranasal Administration (n = 104)
Location of overdose in MSIC facility		
Stage 1 (reception)	1 (1.1)	NA
Stage 2 (injecting booths)	84 (90.3)	93 (89.4)
Stage 3 (recovery space)	8 (8.6)	11 (10.6)
Period		
1st (up to August 6, 2014)	52 (55.9)	61 (58.7)
2nd (from December 8, 2015)	41 (44.1)	43 (41.3)
Drug reported injected		
Heroin	56 (60.2)	65 (62.5)
Pharmaceutical opioids^a^	21 (22.6)	20 (19.2)
Fentanyl	11 (11.8)	14 (13.5)
Methadone	5 (5.4)	5 (4.8)
Concomitant alcohol use^b^		
Yes	20 (23.0)	27 (27.6)
No	67 (77.0)	71 (72.4)
Current pharmacotherapy (methadone or buprenorphine)		
Yes	3 (3.2)	5 (4.8)
No	90 (96.7)	99 (95.2)
**Overdose Attribution**		
Reduced tolerance^c^		
Yes	29 (31.2)	43 (41.3)
No	64 (68.8)	61 (58.7)
Concurrent CNS depressant use		
Yes	61 (65.6)	72 (69.2)
No	32 (34.4)	32 (30.8)
Higher-quality drug		
Yes	12 (12.9)	12 (11.5)
No	81 (87.1)	92 (88.5)
Higher-quantity drug		
Yes	7 (7.5)	10 (9.6)
No	86 (92.5)	94 (90.4)

Abbreviations: CNS, central nervous system; MSIC, Uniting Medically Supervised Injecting Centre; NA, not applicable.

^a^ Pharmaceutical opioids include morphine and oxycodone.

^b^ Reported as having been consumed prior to entering facility with some missing data, so the sample size was 185.

^c^ As described by client during postresuscitation questioning.

For secondary time-to-event outcomes (RR and GCS score), medians and 95% CIs for each study arm were reported as descriptive measures, as were Kaplan-Meier survival plots. Cox proportional hazards regression models, with shared γ frailties to account for within-participant clustering effects^22^ and participants as a random effect, were used to compare between treatment groups. Hazard ratios (HRs) and 95% CIs are reported as treatment effect. We considered the assumption of proportional hazards and explored this assumption through visual inspection of survival and hazard plots.

As clients could be enrolled multiple times, secondary analyses involving only the first overdose of clients were conducted using analogous methods for primary binary (logistic regression) and secondary time-to-event (Cox proportional hazards) outcomes.

All analyses were carried out using Stata, version 15 (StataCorp LLC) by one of us (M.M.). A 2-tailed *P* = .05 indicated statistical significance across all statistical testing.

## Results

### Clients

Almost all 215 eligible opioid overdose cases were enrolled in the trial (Figure 1). Of the 200 cases of opioid overdose enrolled, 197 (98.5%) were included in the analysis sample; 93 (47.2%) were randomized to receive intramuscular naloxone and 104 (52.8%) to receive intranasal naloxone. Data from 3 cases were excluded for final analysis, as intranasal preparation was not successfully administered for 1 case, 1 received additional naloxone before the rescue 10-minute period, and 1 did not receive medication because the individual became alert directly after randomization and before medication could be given. The 197 cases comprised 127 individual clients, of whom 99 presented only once and 28 presented multiple times (12 presented twice; 5, three times; 5, four times; 2, five times; 1, six times; 1, seven times; and 2, eight times) with blinded assignments.

Data for all 197 overdose cases, according to the treatment they received, are shown in Table 1. The 2 groups were similar, consisting of predominantly men (173 [87.8%]) and with a mean (SD) age of 34.0 (7.82) years, reflecting the sex and age composition of the population who typically use the MSIC. Approximately half of the participants (46 [50.5%] in the intramuscular group; 50 [49.0%] in the intranasal group) self-reported having a blood-borne virus. No statistically significant differences were observed between treatment groups at baseline.

### Overdose Characteristics

Characteristics of the overdose events varied little between treatment groups (Table 2). Most overdoses occurred in the injecting booth stage of the MSIC (84 [90.3%] in the intramuscular group; 93 [89.4%] in the intranasal group). Heroin was most frequently reported as the injected opioid (56 [60.2%] in the intramuscular group; 65 [62.5%] in the intranasal group) followed by other pharmaceutical opioids (21 [22.6%] in the intramuscular group; 20 [19.2%] in the intranasal group) and fentanyl (11 [11.8%] in the intramuscular group; 14 [13.5%] in the intranasal group). Few differences were observed between groups regarding presentation in the first or second stage nor the order of administration. In addition, little variation was noted between the 2 groups in reports of concurrent central nervous system depressant use (61 [65.6%] in the intramuscular group; 72 [69.2%] in the intranasal group) as well as reported quality or quantity of opioid. The 2 treatment groups were similar in all other overdose characteristics, with no statistically significant differences. No clients required further treatment outside of the MSIC.

### Efficacy End Points

Clients randomized to intramuscular naloxone administration were less likely to require secondary naloxone compared with those randomized to intranasal naloxone administration (8 [8.6%] vs 24 [23.1%]; OR, 0.35; 95% CI, 0.15-0.66; *P* = .002) (Table 3). This difference means that 7 individuals will need to be given intramuscular naloxone to prevent a rescue dose for 1 additional client (number needed to treat, 6.9; 95% CI, 4.3-17.4). No major adverse events were reported for either group.

**Table 3.** Between-Group Analysis of Main Outcome Measures in All Cases

Outcome	Intramuscular Administration	Intranasal Administration	Intervention Effect (95% CI)
Secondary naloxone, No. (%)			0.35 (0.15-0.66)^a^
Yes	8 (8.6)	24 (23.1)	
No	85 (91.4)	80 (76.9)	
Time to GCS score ≥13, min			1.65 (1.21-2.25)^b^
No.^c^	86	91	
Median (95% CI)	8.0 (6.8-9.2)	15.0 (13.9-16.1)	
Time to RR ≥10, min			1.81 (1.28-2.56)^b^
No.^c^	77	93	
Median (95% CI)	8.0 (6.1-9.9)	17.0 (14.1-19.9)	

Abbreviations: GCS, Glasgow Coma Scale (score range: 3-15, with the highest score indicating normal responses); RR, respiratory rate.

^a^ Odds ratio and 95% CI for dichotomized outcomes.

^b^ Hazard ratio for time-to-event outcomes.

^c^ Some data were missing for these outcomes and were treated as censored observations.

**Figure 2.**
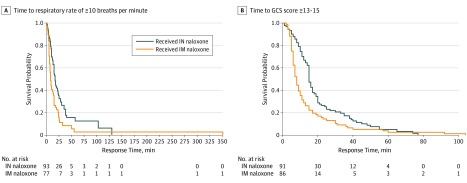
Kaplan-Meier Survival Curve for (A) Time to Respiratory Rate ≥10 and (B) Time to Glasgow Coma Scale (GCS) score ≥13 IM indicates intramuscular; IN, intranasal.

Figure 2 shows the Kaplan-Meier curves of the full range of response times for respiratory rate and GCS score thresholds. The median time to an RR of at least 10 breaths per minute was 8.0 minutes (95% CI, 6.1-9.9) for intramuscular administration, compared with 17.0 minutes (95% CI, 14.1-19.9) for intranasal administration, which equated to an 81% increase in hazard (HR, 1.81; 95% CI, 1.28-2.56; *P* = .001). The median time to adequate GCS score greater than or equal to 13 was 8.0 minutes (95% CI, 6.8-9.2) for intramuscular administration, compared with 15.0 minutes (95% CI, 13.9-16.1) for intranasal administration, which equated to a 65% increase in hazard (HR, 1.65; 95% CI, 1.21-2.25; *P* = .002). Secondary analyses of data from the first presentations for each client (n = 127) produced an almost identical pattern of results (see the eAppendix and eTable in Supplement 2).

## Discussion

In this double-blind, double-dummy randomized clinical trial of 800 μg of intranasal naloxone hydrochloride per 1 mL solution, only 23.1% of cases required a rescue dose of naloxone. However, the same dose of naloxone given intramuscularly was superior in effect. This pattern was evident for the primary outcome, which compared the requirement for a rescue dose of naloxone 10 minutes after baseline and secondary temporal response outcomes.

This study builds on previous trials, which have shown that, although naloxone can be administered intranasally to reverse opioid overdose, the intranasal route of administration is less effective than the intramuscular route when comparable doses and dose concentrations are used.^3,5^ To our knowledge, unlike in previous work, treatment allocation in the present study was hidden from study personnel and researchers through blinding, thus reducing any bias from differential treatment between study arms. Nevertheless, the observed effects on the primary outcome were similar to those observed in previous unblinded trials conducted in the prehospital setting,^3,5^ but the differences were statistically significant in this study. Furthermore, we observed significantly greater times to adequate response in the intranasal condition compared with the intramuscular condition, in contrast to a randomized clinical trial that did not find a significant difference in response^5^ (although the nonsignificant difference observed was in the same direction as the findings in the present trial).

This study was responsive to the practice of intranasal naloxone administration involved in take-home naloxone programs and some paramedic services in which the formulations designed for intramuscular administration are used in conjunction with a nasal atomizer.^6,7,8,23,24^ New pharmacokinetic data suggest that even highly concentrated naloxone doses are absorbed less efficiently when given intranasally compared with doses given intramuscularly.^12^ This outcome suggests that larger doses are needed for intranasal administration (eg, 2 mg per 0.1 mL) to match the plasma levels of 400 μg given intramuscularly (the smallest dose recommended by the World Health Organization for community programs and a typical clinical dose^11,19^), at least in the first 5 minutes after administration.^12^ Larger, more concentrated intranasal doses now feature in products that have been approved by the US Food and Drug Administration (eg, 4 mg per 0.1 mL) and 1 product recently approved by the Australian Therapeutic Goods Administration (2 mg per 0.1 mL). However, whether the actual atomization of these products (that share the same basic design) differs is not clear. Furthermore, the plasma concentrations in these doses of intranasal naloxone continue to rise after the first 5 minutes of administration and remain more than double that of the 400-μg dose given intramuscularly 1 hour after administration.^12^ The effect of this higher plasma concentration on patient safety and treatment experience is unknown.

Intranasal administration of naloxone confers several advantages over intramuscular administration. Intranasal administration removes the possibility of needlestick injury, and intramuscular administration typically requires higher levels of training than intranasal drug delivery, particularly for people unfamiliar with injection (such as noninjecting family members of people who inject drugs). One exception here is a naloxone autoinjector, which is easy to use but is dramatically more expensive than other preparations.^25^

Further research is needed to identify whether the larger doses currently given intranasally can achieve the same effects as the recommended doses administered intramuscularly that have been found in pharmacokinetic studies. Such findings would inform the translation into practice in real-world settings in which opioid overdoses are likely to occur. In addition, the clinical significance of the delayed response to intranasal naloxone administration needs to be determined, particularly given the large number of reversals reported when using a seemingly inferior route of naloxone administration in take-home naloxone programs in the community.^26^ Establishing the optimal dose and concentration of intranasal naloxone to respond to opioid overdose in real-world conditions in a way that balances opioid antagonism and the potential for opioid withdrawal is an international priority,^15^ and supervised injecting facilities offer a suitable environment in which to carry out this work.

### Limitations

This trial has several limitations. First, it included only clients drawn from the MSIC, which is one of a few supervised injecting centers in the world and serves a vulnerable group of clients. However, the characteristics of the clients who experienced overdose were similar to those reported by people who injected drugs in other settings with similar populations.^5,27,28^ Second, the overdose management protocol in the MSIC specifies 5 minutes of ventilation before any naloxone administration. Many clients respond adequately to ventilation alone in that period, and so the trial participants were likely to have experienced relatively severe overdoses. However, resuscitation was commenced as soon as overdose was recognized according to our criteria, which means the participants did not experience the delay between overdose and response that can occur in other settings (eg, when paramedics attend). Third, this study was not designed to examine specific clinical implications of any differences between administration routes, including any differences in the occurrence of needlestick injury. Fourth, most of the overdoses that occurred involved heroin; thus, the applicability of the dosing regimen (in either trial arm) to other more potent opioids, such as illicit fentanyl, for which larger doses of naloxone have been recommended,^29^ is unknown.

## Conclusions

The findings from this double-blind, double-dummy randomized clinical trial suggest that naloxone given intranasally can be effective in reversing opioid overdose. However, intranasal administration was not as effective as a comparable dose at a comparable concentration administered intramuscularly.

## Notes

Supplement 1. **Trial Protocol**

Supplement 2. **eAppendix.** Analysis of First Client Presentations **eTable.** Between Group Analysis of Main Outcome Measures—First Presentations Only

Supplement 3. **Data Sharing Statement**

## References

[1] Boyer EW Management of opioid analgesic overdose. N Engl J Med. 2012;367(2):-. doi:10.1056/NEJMra1202561 22784117 PMC3739053

[2] Barton ED, Ramos J, Colwell C, Benson J, Baily J, Dunn W Intranasal administration of naloxone by paramedics. Prehosp Emerg Care. 2002;6(1):54-58. doi:10.1080/10903120290938797 11789651

[3] Kelly AM, Kerr D, Dietze P, Patrick I, Walker T, Koutsogiannis Z Randomised trial of intranasal versus intramuscular naloxone in prehospital treatment for suspected opioid overdose. Med J Aust. 2005;182(1):24-27. doi:10.5694/j.1326-5377.2005.tb06550.x 15651944

[4] Kerr D, Dietze P, Kelly AM Intranasal naloxone for the treatment of suspected heroin overdose. Addiction. 2008;103(3):379-386. doi:10.1111/j.1360-0443.2007.02097.x 18269360

[5] Kerr D, Kelly AM, Dietze P, Jolley D, Barger B Randomized controlled trial comparing the effectiveness and safety of intranasal and intramuscular naloxone for the treatment of suspected heroin overdose. Addiction. 2009;104(12):2067-2074. doi:10.1111/j.1360-0443.2009.02724.x 19922572

[6] Doe-Simkins M, Walley AY, Epstein A, Moyer P Saved by the nose: bystander-administered intranasal naloxone hydrochloride for opioid overdose. Am J Public Health. 2009;99(5):788-791. doi:10.2105/AJPH.2008.146647 19363214 PMC2667836

[7] Strang J, McDonald R, Tas B, Day E Clinical provision of improvised nasal naloxone without experimental testing and without regulatory approval: imaginative shortcut or dangerous bypass of essential safety procedures? Addiction. 2016;111(4):574-582. doi:10.1111/add.13209 26840916

[8] Walley AY, Doe-Simkins M, Quinn E, Pierce C, Xuan Z, Ozonoff A Opioid overdose prevention with intranasal naloxone among people who take methadone. J Subst Abuse Treat. 2013;44(2):241-247. doi:10.1016/j.jsat.2012.07.004 22980450

[9] Wermeling DP A response to the opioid overdose epidemic: naloxone nasal spray. Drug Deliv Transl Res. 2013;3(1):63-74. doi:10.1007/s13346-012-0092-0 23734342 PMC3668569

[10] Dowling J, Isbister GK, Kirkpatrick CM, Naidoo D, Graudins A Population pharmacokinetics of intravenous, intramuscular, and intranasal naloxone in human volunteers. Ther Drug Monit. 2008;30(4):490-496. doi:10.1097/FTD.0b013e3181816214 18641540

[11] Krieter P, Chiang N, Gyaw S, Pharmacokinetic properties and human use characteristics of an FDA-approved intranasal naloxone product for the treatment of opioid overdose. J Clin Pharmacol. 2016;56(10):1243-1253. doi:10.1002/jcph.759 27145977

[12] McDonald R, Lorch U, Woodward J, Pharmacokinetics of concentrated naloxone nasal spray for opioid overdose reversal: phase I healthy volunteer study. Addiction. 2018;113(3):484-493. doi:10.1111/add.14033 29143400 PMC5836974

[13] Skulberg AK, Tylleskar I, Nilsen T, Pharmacokinetics and -dynamics of intramuscular and intranasal naloxone: an explorative study in healthy volunteers. Eur J Clin Pharmacol. 2018;74(7):873-883. doi:10.1007/s00228-018-2443-3 29568976

[14] Tylleskar I, Skulberg AK, Nilsen T, Skarra S, Jansook P, Dale O Pharmacokinetics of a new, nasal formulation of naloxone. Eur J Clin Pharmacol. 2017;73(5):555-562. doi:10.1007/s00228-016-2191-1 28144724

[15] Skulberg AK, Åsberg A, Khiabani HZ, Røstad H, Tylleskar I, Dale O Pharmacokinetics of a novel, approved, 1.4-mg intranasal naloxone formulation for reversal of opioid overdose-a randomized controlled trial. Addiction. 2019. doi:10.1111/add.14552 30644628

[16] Glaser A, Arakaki D, Chan GM, Hoffman RS Randomised trial of intranasal versus intramuscular naloxone in prehospital treatment for suspected opioid overdose. Med J Aust. 2005;182(8):427; author reply 427, 429. doi:10.5694/j.1326-5377.2005.tb06765.x 15850442

[17] Moher D, Schulz KF, Altman DG The CONSORT statement: revised recommendations for improving the quality of reports of parallel-group randomised trials. Lancet. 2001;357(9263):1191-1194. doi:10.1016/S0140-6736(00)04337-3 11323066

[18] Ambulance Service of New South Wales Protocols and Pharmacology. January 2011. Sydney, Australia: Ambulance Service of New South Wales; 2011.

[19] World Health Organization Community Management of Opioid Overdose. Geneva, Switzerland: World Health Organization; 2014. 25577941

[20] Lewis JA Statistical principles for clinical trials (ICH E9): an introductory note on an international guideline. Stat Med. 1999;18(15):1903-1942. doi:10.1002/(SICI)1097-0258(19990815)18:15<1903::AID-SIM188>3.0.CO;2-F 10440877

[21] McCulloch CE, Neuhaus JM Generalized Linear Mixed Models. Chichester, NY: John Wiley & Sons, Ltd; 2001.

[22] Liu L, Wolfe RA, Huang X Shared frailty models for recurrent events and a terminal event. Biometrics. 2004;60(3):747-756. doi:10.1111/j.0006-341X.2004.00225.x 15339298

[23] Madah-Amiri D, Clausen T, Lobmaier P Rapid widespread distribution of intranasal naloxone for overdose prevention. Drug Alcohol Depend. 2017;173:17-23. doi:10.1016/j.drugalcdep.2016.12.013 28182982

[24] Robinson A, Wermeling DP Intranasal naloxone administration for treatment of opioid overdose. Am J Health Syst Pharm. 2014;71(24):2129-2135. doi:10.2146/ajhp130798 25465584

[25] Wang A, Kesselheim AS Government patent use to address the rising cost of naloxone: 28 U.S.C. § 1498 and Evzio. J Law Med Ethics. 2018;46(2):472-484. doi:10.1177/1073110518782954 30146993

[26] Wheeler E, Davidson PJ, Stephen Jones T, Irwin KS; Centers for Disease Control and Prevention (CDC) Community-based opioid overdose prevention programs providing naloxone–United States, 2010. MMWR Morb Mortal Wkly Rep. 2012;61(6):101-105. https://www.cdc.gov/mmwr/preview/mmwrhtml/mm6106a1.htm. Accessed October 14, 2019. 22337174 PMC4378715

[27] Dietze P, Jolley D, Fry C, Bammer G Transient changes in behaviour lead to heroin overdose: results from a case-crossover study of non-fatal overdose. Addiction. 2005;100(5):636-642. doi:10.1111/j.1360-0443.2005.01051.x 15847621

[28] van Beek I, Kimber J, Dakin A, Gilmour S The Sydney Medically Supervised Injecting Centre: reducing harm associated with heroin overdose. Crit Public Health. 2004;14(4):391-406. doi:10.1080/09581590400027528

[29] Moss RB, Carlo DJ Higher doses of naloxone are needed in the synthetic opiod era. Subst Abuse Treat Prev Policy. 2019;14(1):6. doi:10.1186/s13011-019-0195-4 30777088 PMC6379922

