# Error in Data in the Abstract

**DOI:** 10.1001/jamanetworkopen.2020.6593

**Published:** 2020-04-24

In the Original Investigation titled “Effect of Intranasal vs Intramuscular Naloxone on Opioid Overdose: A Randomized Clinical Trial,”^1^ published online November 13, 2019, in the Results section of the Abstract, the hazard data for respiratory rate and Glasgow Coma Scale score were transposed. This article has been corrected.^1^
