# Supplementary material for: Effect of Intranasal vs Intramuscular Naloxone on Opioid Overdose: A Randomized Clinical Trial
Source: JAMA Netw Open. 2019 Nov 13;2(11):e1914977. doi: 10.1001/jamanetworkopen.2019.14977 (PMC6902775; doi:10.1001/jamanetworkopen.2019.14977)
Supplement: Supplement 1. — Trial Protocol [file jamanetwopen-2-e1914977-s001.pdf]

Supplementary Material

This supplement contains the following items:

1. Original protocol (pp 2-16) & final protocol (pp17-30)

Summary of changes: There were no major changes to the protocol accepted by the thics Committee. The only major change was that the order in which drugs were given (intranasal first, intramuscular second) was reversed halfway through the trial. Otherwise, minor changes to study personnel were noted.

2. Final statistical analysis plan (p31)

Summary of changes: The logic of the analysis plan outlined in the protocol was maintained through to the final analysis. We did however change the analysis to account for clustering of individuals who had been included on multiple occasions as we had not identified this as a possibility at the planning stage. We not only used techniques to account for this clustering but we also conducted a sub-analysis in which we considered only those who had experienced one overdose. As this pattern was almost idnetical we report the overall analysis in the manuscript.

Protocol Title:

Randomised, double-blinded clinical trial comparing IN and IM Naloxone for suspected opioid overdose

Version Number: 2010.4

Date of Protocol: 29/06/11

SYNOPSIS

Protocol title: Randomised, double-blinded clinical trial comparing IN and IM Naloxone for suspected opioid overdose

Protocol version: 2010.4

LIST OF INVESTIGATORS

Chief Investigators:

Dr Marianne Jauncey
Medical Director
Sydney Medically Supervised Injecting Centre (Sydney MSIC)
PO Box 293
Kings Cross NSW 1340
Ph: (02) 9360 0194 Fax: (02) 9360 0707
Email: mjauncey@nchecr.unsw.edu.au

A/Professor Paul Dietze
Head, Alcohol and Other Drugs Research Group
Centre for Population Health,
Macfarlane Burnet Institute for Medical Research and Public Health
GPO Box 2284
Melbourne VICT 3001
Ph: (03) 9282 2111 Fax: (03) 9282 2138
pauld@burnet.edu.au

Associate Investigators:

Dr Debra Kerr
Senior Lecturer
Victoria University
School of Nursing and Midwifery
McKechnie St
St Albans VICT 3021

Dr Allison Salmon
allisons@nswcc.org.au

Ms Collette McGrath
mcgrathco@sesiahs.health.nsw.gov.au

Dr Ingrid van Beek
Medical Director
Kirketon Road Centre
PO Box 22
Kings Cross NSW 1340

Ian Flaherty
Research Co-ordinator
Sydney MSIC
iflaherty@sydneymsic.com
(contact for the project: 9360 1191)

Ms Julie Latimer
Nursing Unit Manager
Sydney MSIC
PO Box 293
Kings Cross NSW 1340

89

Study title: Randomised, double-blinded clinical trial comparing IN and IM Naloxone for suspected opioid overdose

Protocol version: 2010.2

Objectives: The aim of this study is to determine whether IN Naloxone is as effective as IM Naloxone for the treatment of acute opioid overdose.

Study design: Prospective, randomised, double-blinded placebo-controlled clinical trial

Planned sample size: 200

Selection criteria: All clients with symptoms/signs of an opioid overdose requiring Naloxone administration will be eligible for entry. This is based on existing and approved MSIC protocols and clinical criteria for overdose (reduced level of consciousness as measured by the Glasgow Coma Score, pinpoint pupils, respiratory depression and/or reduced oxygen saturations as measured by pulse oximetry). Participants will be prospectively and consecutively enrolled during all operating hours of the service. All new clients of the MSIC will be informed of the study at registration. All current clients will be informed of the study by comprehensive advertisement at the MSIC. Consent for enrolment in the study will be obtained only once, so that clients can be included in the study during any subsequent use of the facility in the case of opioid overdose.

Study procedure: MSIC staff will manage drug overdoses using existing clinical protocols.[1] These state that a client will receive airway management and oxygenation either via Hudson mask or artificial ventilation (bagging) for five minutes before being considered in need of Naloxone. Where a client's response after five minutes is inadequate, Naloxone will be administered by a registered nurse in accordance with existing standing orders from the Medical Director.

Inadequate response will be defined as:

- Glasgow Coma Score < 13
- pinpoint pupils
- respirations < 10/min and/or
- reduced oxygen saturations (< 95%)

Study participants will receive a random allocation of Naloxone in one of 2 forms:

1. Active intranasal Naloxone and intramuscular placebo, or
2. Intranasal placebo and active intramuscular Naloxone

Statistical considerations:

Recently, the study findings of a RCT comparing IN and IM Naloxone have been reported.[2] In that study, requirement for a secondary dose of Naloxone was reported as IN: 18% vs. IM: 4%. We estimate (using JavaStat) that we would need

---

[1] Decreased level of consciousness – Sydney MSIC Clinical Policy and Procedure 1.16.

[2] Kerr D, Dietze P, Kelly AM. Intranasal Naloxone for the treatment of suspected heroin overdose. Review article. *Addiction*. 2008 Mar: 103(3) 379-86

a sample size of 99 per group to achieve adequate power (power=0.8, with 95% confidence) to detect a difference of 14% in requirement for secondary Naloxone.

The project aims to recruit approximately 200 participants, and all costing is based on this. At current rates of opioid overdose and Naloxone administration (based on the most recent quarter) it is anticipated that 180 cases will be enrolled within 12-18 months. After six months rate of recruitment will be reviewed to assess expected time lines.

TABLE OF CONTENTS

BACKGROUND
- Disease Background
- Rationale for Performing the Study

STUDY OBJECTIVES
- Primary Objective
- Secondary objectives

STUDY DESIGN
- Design
- Study Groups
- Number of Participants
- Number of Centres
- Duration

PARTICIPANT SELECTION
- Inclusion Criteria
- Exclusion Criteria

STUDY OUTLINE
- Study Flow Chart
- Investigation Plan
- Recruitment and Screening
- Informed Consent Process
- Enrolment Procedure
- Randomisation Procedure
- Study Procedures

SAFETY
- Adverse Event Reporting
- Serious Adverse Event Reporting
- Data Safety and Monitoring Board - membership and responsibilities
- References to national and international guidelines on research in humans
- Early Study Termination

BLINDING AND UNBLINDING

STATISTICAL CONSIDERATIONS
- Sample Size Calculation

Analysis Plan

STORAGE AND ARCHIVING OF STUDY DOCUMENTS

REFERENCES

APPENDICES

Data Collection Sheets / Case Report Forms

Questionnaires

[Please note all sections may not be relevant to your study design delete / include as necessary, the sections marked with * are essential for all studies]

1. BACKGROUND

1.1. DISEASE BACKGROUND*

Drug overdose is a leading cause of death among people who inject drugs. The Sydney MSIC was established in 2001 with the primary aim of reducing the morbidity and mortality of drug overdoses in the local area. In nine years, the MSIC has treated over 3000 acute opioid overdoses on site without a single fatality. Overdose management protocols are in place for nursing staff to administer intramuscular Naloxone, as well as provide external airways resuscitation and oxygen. Approximately 20% of all heroin/opioid overdoses (approximately 450 cases to date) have required the administration of Naloxone. Due to the high frequency of opioid overdose routinely treated onsite, and the experienced staff who manage them, the MSIC provides a unique and ideal setting to investigate alternative Naloxone administration routes.

IM and intravenous Naloxone are currently the standard modes of emergency treatment for an opioid overdose, however pharmacology data indicate that Naloxone is 100% bioavailable through the nasal mucosa.[1] Naloxone can be sprayed into the nose via a mucosal atomisation device and evidence indicates that IN Naloxone may not only be as effective a route of administration as IM administration, but also a practical and realistic alternative.[2-4] There have been a number of previous studies examining IN Naloxone.[2-13] These have generally been cohort studies[7-11] with only two previous randomised trials[12-13] neither of which were double-blinded. The first randomised trial used a large volume of fluid for intranasal administration which may have limited efficacy.[12] The subsequent trial showed a slightly higher rate of ‘rescue’ Naloxone given to those patients whose initial dose was delivered intranasally[13]. The authors felt that this may have been due to the non blinded nature of the study, with the additional dose being a subjective decision dependent of individual paramedic’s comfort in waiting for a response.

1.2. RATIONALE FOR PERFORMING THE STUDY*

The aim of this study is to determine whether Naloxone, an opioid reversal agent, is as effective for the treatment of acute opioid overdose when administered via the intranasal route compared with the intramuscular route. The term 'opioid' refers to a class of drugs that includes heroin, and the prescription medications oxycontin and morphine. Injection of Naloxone into a muscle is currently the standard method of emergency treatment for an opioid overdose on site at MSIC. However, Naloxone can be administered as an intranasal (IN) spray using a 'mucosal atomisation device'. Evidence suggests that IN Naloxone may be an effective and practical alternative. The significant benefits of IN administration include removing the risk of needlestick injury (and thus any blood borne virus transmission risk) to

treating personnel. This is particularly relevant to ambulance officers and paramedics, as well as other emergency health care workers, such as in the hospital emergency department setting.

Additionally, given the ease of administration and reduced issues regarding disposal of used needles, the availability of IN Naloxone may potentially be extended. This may include non health care workers who regularly deal with opioid overdose in community settings, and drug users' peers, who are likely to be present in the event of an overdose. The Sydney Medically Supervised Injecting Centre (MSIC) provides the ideal setting for this study.

2. STUDY OBJECTIVES*

2.1. PRIMARY OBJECTIVE*

The aim of this study is to determine whether IN Naloxone is as effective as IM Naloxone for the treatment of an acute opioid overdose.

3. STUDY DESIGN*

3.1. DESIGN*

Prospective, randomised, double-blinded, placebo-controlled clinical trial

3.2. NUMBER OF PARTICIPANTS*

Approximately 200

3.3. NUMBER OF CENTRES

Single-site study

3.4. DURATION

- NOVEMBER 2010 – OCTOBER 2012 (MAX.)
- Participants will be consecutively enrolled during this period. We estimate at current rates of overdose requiring Naloxone, eighteen months to two years will be required to achieve a sample of 200 participants.

4. PARTICIPANT SECTION

4.1. INCLUSION CRITERIA*

For example but not limited to:

* Sex: male and female
* Age range: aged 18 and over
* Weight: not defined
* Height: not defined
* Disease status: All clients with symptoms/signs of an opioid overdose requiring Naloxone administration will be eligible for entry. This is based on existing and approved MSIC protocols and clinical criteria for overdose (reduced level of consciousness as measured by the Glasgow Coma Score, pinpoint pupils, respiratory depression and/or reduced oxygen saturations as measured by pulse oximetry).

4.2. Exclusion Criteria*

The research design specifically excludes pregnant women, the human foetus and individuals under the age of eighteen. Sydney MSIC specifically excludes these individuals by the following procedure:

1. If it is suspected by MSIC staff that a client may be pregnant, they are referred to a local drug and alcohol programme where they undergo a pregnancy test. The results are then returned to MSIC.
2. If it is suspected a potential client may be under eighteen years old, formal ID is requested of the potential client by MSIC staff. If the person is over eighteen, a copy of the ID is kept on file. If the person is under eighteen, they are referred to a local drug and alcohol service.
3. Anyone who presents as already intoxicated from alcohol or other drugs, anyone accompanied by a child and anyone who has not previously injected drugs are already excluded from using the MSIC according to the internal management protocols required by legislation.
4. Probable incidental recruitment may include people whose primary language is other than English, people in existing dependent or unequal relationships, people with a cognitive impairment, intellectual disability or a mental illness and people from Aboriginal and Torres Strait Islander backgrounds.

5. STUDY OUTLINE*

5.1. Study Flow Chart

Diagram of the study design:

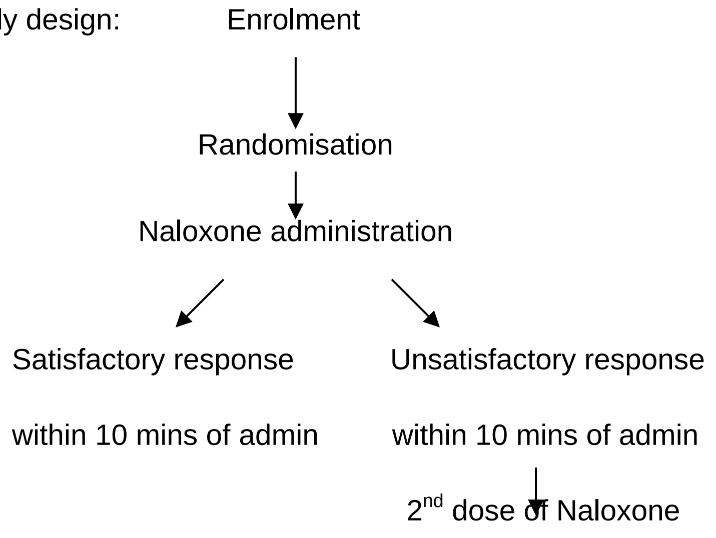

5.2. Investigation Plan*

Enrolment Visit	Client 1	Client 2	Client 3	Client …	Client 200
Informed Consent	✓	✓	✓	✓	✓
Inclusion / Exclusion criteria	✓	✓	✓	✓	✓
Narcan Administration	✓	✓	✓	✓	✓

Vital signs collected	✓	✓	✓	✓	✓
Adverse Event & Serious Adverse Event Assessment	✓	✓	✓	✓	✓

5.3. Study Procedure Risks*

There are no additional risks for the clients participating in this study outside the risks that usually accompany the administration of Naloxone. The risks usually involved with administering Naloxone include abrupt reversal of opioid sedation and precipitated withdrawal in an opiate-dependent individual. This may include nausea, vomiting, sweating, tachycardia, increased blood pressure, hyperventilation, tremulousness. These risks are mitigated by access to immediate medical attention, including oxygenation. Additionally, the smaller dose of Naloxone (800mcg) given by MSIC nursing staff causes less risk of precipitated opiate withdrawal than the larger dose given by ambulance officers and paramedics (2mg).

There are rare instances in the medical literature of seizures, ventricular tachycardia and fibrillation, pulmonary oedema and cardiac arrest during abrupt post-operative reversal of opioid depression by Naloxone. The pathogenesis of these effects are not clear; however, it is worth noting that none of these complications have ever been observed at MSIC.

It is important to note that in the event of inadequate oxygen saturations as monitored by pulse oximetry, at all times, nurses have the capacity under existing protocols to intervene with Naloxone before five minutes have expired

In addition, the only difference between the 2 study groups is the route of administration. Previous studies have shown that there is no greater risk with Intranasal administration compared with IM Naloxone. In fact, the 1st study by Kelly et al (2005) showed a reduced risk of adverse events for the IM group.

5.4. Recruitment and Screening*

- Recruitment: all MSIC clients are potential participants for this study, as all MSIC clients may potentially experience opioid overdose while using the centre. Flyers will be placed around MSIC notifying clients of the study.

- Screening: All clients with symptoms/signs of an opioid overdose requiring Naloxone administration will be eligible for the study. This is based on existing and approved MSIC protocols and clinical criteria for overdose:

- Glasgow Coma Score < 13
- pinpoint pupils
- respirations < 10/min and/or
- reduced oxygen saturations (< 95%)

5.5. Informed Consent Process*

We have used Section 2.2.5 of the *National Statement on Ethical Conduct in Human Research* to guide us in a method of obtaining oral consent:

Informed consent will be obtained from the participants in the following way:

- By ensuring that the trial is comprehensively advertised at MSIC;
- Any newly registering clients will be provided details of the trial;
- By asking the clients once if they consent to participate and offer any further information requested;
- When a client declines to participate this will be flagged on the database. This list of clients will be regularly reviewed at clinical case reviews so that all staff are familiar with the clients who do not want to participate;
- If a consenting client is enrolled, they will be informed of their participation after the intervention. At a point that is suitable bearing in mind the process of the client's recovery, they will be asked if they wish to withdraw that event's data from the study.

5.6. ENROLMENT PROCEDURE*

The participant will be enrolled into the study after the informed consent process has been completed and the participant has met all inclusion criteria and none of the exclusion criteria. Participants will be consecutively enrolled during all operating hours of the service. All clients of the MSIC will be informed of the study at registration. The participant will be informed of their enrollment once they have fully regained consciousness. If they decline to participate, the data will be eliminated from the database.

5.7. RANDOMISATION PROCEDURE

MSIC staff will manage drug overdoses using existing clinical protocols. These state that a client will receive airway management and oxygenation either via a Hudson mask or artificial ventilation (bagging) for five minutes before being assessed as to the need for Naloxone. Where a client's response after five minutes is inadequate, Naloxone will be administered by a registered nurse in accordance with existing standing orders from the Medical Director.

Study participants will receive a random allocation of Naloxone in one of 2 forms:

a. Active intranasal Naloxone and intramuscular placebo, or
b. Intranasal placebo and active intramuscular Naloxone

Study packs will be pre-prepared and contain two clearly labelled vials – 1) Intranasal - Naloxone 800mcg/1mL or placebo (1mL) for IN administration and 2) Intramuscular - 800mcg/1ml or placebo for IM administration. The vials will be labelled as intranasal and intramuscular, therefore the person administering the drugs will be blinded to the treatment arms. As per MSIC protocol, two staff members will attend the patient during treatment of opioid overdose. A third attending staff member will record the participants' vital signs on a chart. The chart is currently in use for overdoses.

The intranasal administration will always precede the intramuscular administration. Contents of the vial for IN administration will be drawn into one syringe. Staff will attach the syringe to a MAD, and administer 0.5mL (400mcg) into each nostril, depressing the syringe rapidly to achieve adequate atomisation. In this way, each study participants will receive 0.5mL of fluid in each nostril. Supportive care/oxygenation will be administered simultaneously, in accordance with existing MSIC clinical protocols

Administration of the IM injection will be by standard practice of drawing the full 1ml into a single 5ml syringe and administering to the deltoid muscle via a 21g needle.

Any client who fails to respond adequately after ten minutes will be eligible for a second dose. This dose will be a known dose of 800mcg of Naloxone administered intramuscularly and not subject to randomisation. In this manner,

all overdoses will receive the same clinical care they would have otherwise, under existing approved clinical protocols, with Naloxone administered at the same time point, in response to the same clinical criteria. The only exception will be that the need for any additional dose of Naloxone will be assessed at ten minutes post-first dose as opposed to five minutes. Importantly, all clients are adequately oxygenated and fully monitored during this time.

Post analysis, the treatment allocations will be de-coded.

The preparation of Naloxone and placebo will be manufactured by a registered pharmaceutical company, Sypharma Pty Ltd, and will comply with national medication quality and safety standards. The complete study packs will be packed by Pharmpackpro Pty Ltd.

6.SAFETY*

6.1. ADVERSE EVENT REPORTING*

In this study an adverse event is defined as: any untoward medical occurrence in a participant which may or may not have a causal relationship with the study treatment. An adverse event can therefore be any unfavorable or unintended sign, symptom or condition and/or an observation that may or may not be related to the study treatment. A register of adverse events will be kept and will be reviewed by the research team fortnightly.

6.2. SERIOUS ADVERSE EVENT REPORTING

In this study a serious adverse event is defined as: any untoward medical occurrence that results in the following: death, is life-threatening, requires inpatient hospitalisation, persistent or significant disability/incapacity, or a condition requiring medical or surgical intervention, such as cardiac arrest, acute pulmonary oedema, cardiac arrhythmias, epileptic seizure. A register of serious adverse events will be kept and reviewed by the research team fortnightly.

6.3. DATA SAFETY AND MONITORING BOARD

The data safety and monitoring board (DSMB) comprises two independent emergency medicine clinicians, Professor Anne-Maree Kelly and Dr David Krieser, employed by Western Health. Both are registered consultants in emergency medicine and both have knowledge of the first two intranasal Naloxone trials at Western Health.

The DSMB will receive a fortnightly update of study enrollments that will include a comparison of requirement for rescue Naloxone for the two groups (intranasal and intramuscular) and any adverse events. All major adverse events (cardiac arrest, acute pulmonary oedema, cardiac arrhythmias, epileptic seizure) will be reported within 24 hours to the DSMB. (Please see note in “Blinding and Unblinding” regarding coding of the treatment arms).

6.4. EARLY TERMINATION

The DSMB may advise the trial be prematurely discontinued if concern arises about differences in adverse event or response rates between the two study arms (intranasal and intramuscular).

7. BLINDING AND UNBLINDING

Blinding will occur in the following way: study packs will be prepared by Pharmpackpro Pty Ltd. Two vials will be in the study packs, one containing the active Naloxone and one containing placebo. Vials will be individually and clearly labeled either ‘intramuscular’ or ‘intranasal’. During the course of the trial only Pharmpackpro Pty Ltd. will know which packs contain either active Naloxone or placebo, intramuscular or intranasal. Pharmpackpro Pty Ltd. will provide the research co-ordinator, Ian Flaherty, with a code assigned to each treatment arm but

that does not reveal whether the arm contains the active Naloxone or placebo. In this way, the two treatment arms will be distinguishable for fortnightly analysis, but the researchers will remain blinded. The packs will be sequentially numbered, and coded according to treatment arm, and the pack number and code used during the trial will be recorded, along with the other clinical observations, by the MSIC staff on the data-form. In this way, the participants, the nurses administering the Naloxone and the researchers will be blinded to the two treatment arms.

Unblinding will occur in the following way: once all participants have been recruited, and the active phase of the trial has concluded, clinically-significant differences in the responses of the participants will be analysed. After this analysis, treatment allocations will be de-coded by obtaining from Pharmpackpro Pty Ltd. the numbers of the packs and codes that correspond to the two treatment arms, thereby revealing to which treatment arm each of the participants belongs.

8. STATISTICAL CONSIDERATIONS*

Recently, the study findings of a RCT comparing IN and IM Naloxone have been reported.[13] In that study, requirement for a secondary dose of Naloxone was reported as IN:18% vs IM:4%. We estimate (using JavaStat) that we would need a sample size of 99 per group to achieve adequate power (power=0.8, with 95% confidence) to detect a difference of 14% in requirement for secondary Naloxone.

Descriptive analyses (proportion, mean, median, effect size difference with 95% confidence intervals) will be conducted to compare groups (IN and IM) for observed differences (demographic data, drug use, alcohol use). Primary outcomes will be compared by univariate analysis including odds ratio with 95% CI, hazard ratio (HR) and chi-square analysis. Multivariate analyses will be conducted (logistic regression, Cox regression). Response time will be compared using a Kaplan-Meier survival curve. A clinically significant difference in response time will be defined as two minutes.

9. STORAGE AND ARCHIVING OF STUDY DOCUMENTS*

Study documents, both hard and soft copies will be stored in accordance with the Australian Code for the Responsible Conduct of Research. The data will be retained securely for the full fifteen years recommended by the code.

Data-forms will be secured in a locked filing cupboard in a locked office at the MSIC. Only the research team will have access to this filing cabinet.

Data will be entered into an electronic database that will be password protected. Only study investigators will have access to the database.

Data will be kept for a period of 15 years after publication of study findings in a peer-reviewed medical journal. At that time, paper files will be destroyed by paper shredder and electronic files will be deleted from computers.

10. REFERENCES*

1. Hussain A, Kimura R, Huang C. Nasal absorption of Naloxone and buprenorphine in rats. *Int J Pharm* 1984; 21:233.

2. Loimer N, Hofmann P, Chaudry H. Nasal administration of Naloxone is as effective as the intravenous route in opiate addicts. *Int J Addic*. 1994 Apr; 29 (6) 819-827.
3. Kerr D, Dietze P, Kelly AM. Intranasal Naloxone for the treatment of suspected heroin overdose. Review article. *Addiction*. 2008 Mar: 103(3) 379-86
4. Ashton H, Hassan Z. Best evidence topic report. Intranasal Naloxone in suspected opioid overdose. *Emerg Med J* 2006; 23: 221-223
5. Wolfe T.R., Bernstone T. Intranasal drug delivery: an alternative to intravenous administration in selected emergency cases. *J Emerg Nurs* 2004; 30(2): 141-7.
6. Costantino H.R., Illum L., Brandt G., Johnson P.H., Quay S.C. Intranasal delivery: physicochemical and therapeutic aspects. *Int J Pharm* 2007; 337(1-2): 1-24.
7. Loimer N., Hofmann P., Chaudhry H.R. Nasal administration of Naloxone for detection of opiate dependence. *J Psychiatr Res* 1992; 26(1): 39-43.
8. Barton E.D., Colwell C.B., Wolfe T., Fosnocht D., Gravitz C., Bryan T., et al. Efficacy of intranasal Naloxone as a needleless alternative for treatment of opioid overdose in the prehospital setting. *J Emerg Med* 2005; 29(3): 265-71.
9. Barton E.D., Ramos J., Colwell C., Benson J., Baily J., Dunn W. Intranasal administration of Naloxone by paramedics. *Prehosp Emerg Care* 2002; 6(1): 54-8.
10. Kelly A.M., Koutsogiannis Z. Intranasal Naloxone for life threatening opioid toxicity. *Emerg Med J* 2002; 19(4): 375.
11. Robertson T., Hendey G., Stroh G., Shalit M. Prehospital intranasal versus intravenous administration of Naloxone for narcotic overdose. *Society for Academic Emergency Medicine*; 2005; New York; 2005. p. 166-7.
12. Kelly A. M., Kerr D., Dietze P., Patrick I., Walker T., Koutsogiannis Z. Randomised trial of intranasal versus intramuscular Naloxone in prehospital treatment for suspected opioid overdose. Med J Aust 2005; 182: 24–7.
13. Kerr, D, Kelly, AM, Dietze, Paul, Jolley, D., Barger, B. IHRA 2008. Annual Conference Oral Presentation. Randomised controlled trial comparing the effectiveness and safety of intranasal and intramuscular Naloxone for the treatment of heroin overdose

Decreased level of consciousness – Sydney MSIC Clinical Policy and Procedure 1.16.

11. APPENDICES

1. Advertisement(s): please see appended PICF
2. Data collection sheet / Case Report Form: please see appended data collection form

Final Protocol

Randomised, double-blind clinical trial comparing IN and IM Naloxone for suspected opioid overdose

Version Number: 2011.1

Date of Protocol: 05/10/2011

LIST OF INVESTIGATORS

Professor Paul Dietze

Head, Alcohol and Other Drugs Research Group; Centre for Population Health, Macfarlane Burnet Institute for Medical Research and Public Health;

GPO Box 2284, Melbourne VIC 3001

Mr Ian Flaherty

Research Coordinator; Sydney MSIC

PO Box 293; Kings Cross NSW 1340

Dr Marianne Jauncey
Medical Director; Sydney Medically Supervised Injecting Centre (Sydney MSIC)
PO Box 293; Kings Cross NSW 1340
A/Professor Debra Kerr
Nursing and Midwifery; Deakin University
1 Gheringap St, Geelong, Vic, 3220
Ms Julie Latimer
Nursing Unit Manager
Sydney MSIC
PO Box 293; Kings Cross NSW 1340
Dr Mohammadreza Mohebbi
Research Fellow
Faculty of Health, Deakin Unversity
Burwood Highway, Burwood VIC
Dr Allison Salmon
Sydney MSIC
PO Box 293; Kings Cross NSW 1340
Dr Ingrid van Beek
Medical Director; Kirketon Road Centre
PO Box 22; Kings Cross; NSW 1340

Randomised, double-blind clinical trial comparing IN and IM Naloxone for suspected opioid overdose

1. BACKGROUND

1.1 DISEASE BACKGROUND

Drug overdose is a leading cause of death among people who inject drugs. The Sydney Medically Supervised Injecting Clinic (MSIC) was established in 2001 with the primary aim of reducing the morbidity and mortality of drug overdoses in the local area. Within the first nine years of opening, the MSIC had treated over 3000 acute opioid overdoses on site without a single fatality. Overdose management protocols are in place for nursing staff to administer intramuscular Naloxone, as well as provide external airways resuscitation and oxygen, for clients who overdose within the MSIC . Due to the high frequency of opioid overdose routinely treated onsite, and the experienced staff who manage them, the MSIC provided a unique and ideal setting to investigate alternative Naloxone administration routes.

IM and intravenous Naloxone are currently the standard modes of emergency treatment for an opioid overdose, however pharmacology data indicate that Naloxone is 100% bioavailable through the nasal mucosa.[1] Naloxone can be sprayed into the nose via a mucosal atomisation device and evidence indicates that IN Naloxone may not only be as effective a route of administration as IM administration, but also a practical and realistic alternative.[2-4] There have been a number of previous studies examining IN Naloxone.[2-13] These have generally been cohort studies[7-11] with only two previous randomised trials[12-13] neither of which were double-blinded.

The first randomised trial used a large volume of fluid for intranasal administration which may have limited efficacy[12]. The subsequent trial showed a slightly higher rate of 'rescue' Naloxone given to those patients whose initial dose was delivered intranasally[13]. The authors felt that this may have been due to the non blinded nature of the study, with the additional dose being a subjective decision dependent of individual paramedic's comfort in waiting for a response.

1.2 RATIONALE FOR PERFORMING THE STUDY

The aim of this study is to determine whether Naloxone, an opioid reversal agent, is as effective for the treatment of acute opioid overdose when administered via the intranasal route compared with the intramuscular route. The term 'opioid' refers to a class of drugs that includes heroin, and the prescription medications oxycontin and morphine. Injection of Naloxone into a muscle is currently the standard method of emergency treatment for an opioid overdose on site at MSIC. However, Naloxone can be administered as an intranasal (IN) spray using a 'mucosal atomisation device'. Evidence suggests that IN Naloxone may be an effective and practical alternative. The significant benefits of IN administration include removing the risk of needlestick injury (and thus any blood borne virus transmission risk) to treating personnel. This is particularly relevant to ambulance officers and paramedics, as well as other emergency health care workers, such as in the hospital emergency department setting.

Additionally, given the ease of administration and reduced issues regarding disposal of used needles, the availability of IN Naloxone may potentially be extended. This

may include non health care workers who regularly deal with opioid overdose in community settings, and drug users' peers, who are likely to be present in the event of an overdose.

2 STUDY OBJECTIVES

2.1 PRIMARY OBJECTIVE

- The aim of this study is to determine whether IN Naloxone is as effective as IM Naloxone for the treatment of an acute opioid overdose.

3 STUDY DESIGN

3.1 DESIGN

- Prospective, randomised, double-blinded, placebo-controlled clinical trial.

3.2 NUMBER OF PARTICIPANTS

- Approximately 200

3.3 NUMBER OF CENTRES

- Single-site study

3.4 DURATION

- November 2010 – October 2012
- Participants will be consecutively enrolled during this period. We estimate at current rates of overdose requiring Naloxone, eighteen months to two years will be required to achieve a sample of 200 participants.

4 PARTICIPANT SELECTION

4.1 INCLUSION CRITERIA

- Age range: 18 years and over
- Weight: not defined
- Height: not defined
- Disease status: All clients with symptoms/signs of an opioid overdose requiring Naloxone administration will be eligible for entry. This is based on existing and approved MSIC protocols and clinical criteria for overdose (reduced level of consciousness as measured by the Glasgow Coma Score, pinpoint pupils, respiratory depression and/or reduced oxygen saturations as measured by pulse oximetry).

4.2 EXCLUSION CRITERIA

The research design specifically excludes pregnant women, the human foetus and individuals under the age of eighteen. Sydney MSIC specifically excludes these individuals from utilising the MSIC by the following procedure:

- If it is suspected by MSIC staff that a client may be pregnant, they are referred to a local drug and alcohol programme where they undergo a pregnancy test. The results are then returned to MSIC.
- If it is suspected a potential client may be under eighteen years old, formal ID is requested of the potential client by MSIC staff. If the person is over eighteen, a copy of the ID is kept on file. If the person is under eighteen, they are referred to a local drug and alcohol service.
- Anyone who presents as already intoxicated from alcohol or other drugs, anyone accompanied by a child and anyone who has not previously injected drugs are already excluded from using the MSIC according to the internal management protocols required by legislation.
- Probable incidental recruitment may include people whose primary language is other than English, people in existing dependent or unequal relationships, people with a cognitive impairment, intellectual disability or a mental illness and people from Aboriginal and Torres Strait Islander backgrounds.

5 STUDY OUTLINE

5.1 STUDY FLOW CHART

Enrolment

↓

Randomisation

↓

Naloxone administration

↙ ↘

Satisfactory response within 10 mins of admin

Unsatisfactory response within 10 mins of admin

↓

2nd dose of Naloxone

5.2 INVESTIGATION PLAN

Enrolment Visit	Client 1	Client 2	Client 3	Client …	Client 200
Informed Consent	✓	✓	✓	✓	✓
Inclusion / Exclusion criteria	✓	✓	✓	✓	✓
Narcan Administration	✓	✓	✓	✓	✓
Vital signs collected	✓	✓	✓	✓	✓
Adverse Event & Serious Adverse Event Assessment	✓	✓	✓	✓	✓

5.3 STUDY PROCEDURE RISK

- There are no additional risks for the clients participating in this study outside the risks that usually accompany the administration of Naloxone. The risks usually involved with administering Naloxone include abrupt reversal of opioid sedation and precipitated withdrawal in an opiate-dependent individual. This may include nausea, vomiting, sweating, tachycardia, increased blood pressure, hyperventilation, tremulousness. These risks are mitigated by access to immediate medical attention, including oxygenation. Additionally, the smaller dose of Naloxone (800mcg) given by MSIC nursing staff causes less risk of

precipitated opiate withdrawal than the larger dose given by ambulance officers and paramedics (2mg).

- There are rare instances in the medical literature of seizures, ventricular tachycardia and fibrillation, pulmonary oedema and cardiac arrest during abrupt post-operative reversal of opioid depression by Naloxone. The pathogenesis of these effects are not clear; however, it is worth noting that none of these complications have ever been observed at MSIC.
- It is important to note that in the event of inadequate oxygen saturations as monitored by pulse oximetry at all times, nurses have the capacity under existing protocols to intervene with Naloxone before five minutes have expired.
- In addition, the only difference between the 2 study groups is the route of administration. Previous studies have shown that there is no greater risk with Intranasal administration compared with IM Naloxone. In fact, the 1st study by Kelly et al (2005) showed a reduced risk of adverse events for the IM group.

5.4 RECRUITMENT AND SCREENING

- Recruitment: all MSIC clients are potential participants for this study, as all MSIC clients may potentially experience opioid overdose while using the centre. Flyers will be placed around MSIC notifying clients of the study.
- Screening: All clients with symptoms/signs of an opioid overdose requiring Naloxone administration will be eligible for the study. This is based on existing and approved MSIC protocols and clinical criteria for overdose include:
    - glasgow coma score < 13
    - pinpoint pupils
    - respirations < 10/min and/or
    - reduced oxygen saturations (< 95%).

5.5 INFORMED CONSENT PROCESS

We have used Section 2.2.5 of the National Statement on Ethical Conduct in Human Research to guide us in a method of obtaining oral consent. Informed consent will be obtained from the participants in the following way:

- By ensuring that the trial is comprehensively advertised at MSIC;
- Any newly registering clients will be provided details of the trial;
- By asking the clients once if they consent to participate and offer any further information requested;
- When a client declines to participate this will be flagged on the database. This list of clients will be regularly reviewed at clinical case reviews so that all staff are familiar with the clients who do not want to participate;
- If a consenting client is enrolled, they will be informed of their participation after the intervention. At a point that is suitable, bearing in mind the process of the client's recovery, they will be asked if they wish to withdraw that event's data from the study.

5.6 ENROLMENT PROCEDURE

The participant will be enrolled into the study after the informed consent process has been completed and the participant has met all inclusion criteria and none of the exclusion criteria. Participants will then be consecutively enrolled during all operating hours of the service. All clients of the MSIC will be informed of the study

at registration. The participant will be informed of their enrollment once they have fully regained consciousness. If they decline to participate, the data will be eliminated from the database.

5.7 RANDOMISATION PROCEDURE

MSIC staff will manage drug overdoses using existing clinical protocols. These state that a client will receive airway management and oxygenation either via a Hudson mask or artificial ventilation (bagging) for five minutes before being assessed as to the need for Naloxone. Where a client's response after five minutes is inadequate, Naloxone will be administered by a registered nurse in accordance with existing standing orders from the Medical Director.

Study participants will receive a random allocation of Naloxone in one of 2 forms:

- Active intranasal Naloxone and intramuscular placebo, or
- Intranasal placebo and active intramuscular Naloxone.

Study packs will be pre-prepared and contain two clearly labelled vials – 1) Intranasal - Naloxone 800mcg/1mL or placebo (1mL) for IN administration and 2) Intramuscular - 800mcg/1ml or placebo for IM administration. The vials will be labelled as intranasal and intramuscular, therefore the person administering the drugs will be blinded to the treatment arms. As per MSIC protocol, two staff members will attend the patient during treatment of opioid overdose. A third attending staff member will record the participants' vital signs on a chart. The chart is currently in use for overdoses.

To minimise potential bias, the order in which the drug will be administered will be rotated approximately midway through the study. Initially the intranasal drug preparation will be administered first, followed by the intramuscular drug preparation. Approximately midway through recruitment (e.g., after 100 enrolments), the intramuscular drug preparation will be administered first, followed by the intranasal drug preparation.

Contents of the vial for IN administration will be drawn into one syringe. Staff will attach the syringe to a MAD, and administer 0.5mL (400mcg) into each nostril, depressing the syringe rapidly to achieve adequate atomisation. In this way, each study participants will receive 0.5mL of fluid in each nostril. Supportive care/oxygenation will be administered simultaneously, in accordance with existing MSIC clinical protocols

Administration of the IM injection will be by standard practice of drawing the full 1ml into a single 5ml syringe and administering to the deltoid muscle via a 23g needle.

Any client who fails to respond adequately after ten minutes will be eligible for a second dose. This dose will be a known dose of 800mcg of Naloxone administered intramuscularly and not subject to randomisation. In this manner, all overdoses will receive the same clinical care they would have otherwise, under existing approved clinical protocols, with Naloxone administered at the same time point, in response to the same clinical criteria. The only exception will be that the need for any additional dose of Naloxone will be assessed at ten minutes post- first dose as opposed to five minutes. Importantly, all clients will be adequately oxygenated and fully monitored during this time.

Post analysis, the treatment allocations will be de-coded.

The preparation of Naloxone and placebo will be manufactured by a registered pharmaceutical company, Sypharma Pty Ltd, and will comply with national medication quality and safety standards. The complete study packs will be packed by Pharmpackpro Pty Ltd.

6. SAFETY

6.1 ADVERSE EVENT REPORTING

In this study an adverse event is defined as any untoward medical occurrence in a participant which may or may not have a causal relationship with the study treatment. An adverse event can therefore be any unfavorable or unintended sign, symptom or condition and/or an observation that may or may not be related to the study treatment. A register of adverse events will be kept and will be reviewed by the research team fortnightly.

6.2 SERIOUS ADVERSE EVENT REPORTING

In this study a serious adverse event is defined as any untoward medical occurrence that results in the following: death, is life-threatening, requires inpatient hospitalisation, persistent or significant disability/incapacity, or a condition requiring medical or surgical intervention, such as cardiac arrest, acute pulmonary oedema, cardiac arrhythmias, epileptic seizure. A register of serious adverse events will be kept and reviewed by the research team fortnightly.

6.3 DATA SAFETY AND MONITORING BOARD

A data safety and monitoring board (DSMB) will comprise two independent and registered emergency medicine clinicians, employed within a major hospital health service in Melbourne, Victoria. Both are emergency medicine consultants.

The DSMB will receive a fortnightly update of study enrollments that will include any adverse events. All major adverse events (cardiac arrest, acute pulmonary oedema, cardiac arrhythmias, epileptic seizure) will be reported within 24 hours to the DSMB. (Please see note in “Blinding and Unblinding” regarding coding of the treatment arms).

6.4 EARLY TERMINATION

The DSMB may advise the trial be prematurely discontinued if concern arises about adverse event rates or delayed response rates.

7. BLINDING AND UNBLINDING

Blinding will occur in the following way: study packs will be prepared by Pharmpackpro Pty Ltd. Two vials will be in the study packs, one containing the active Naloxone and one containing placebo. Vials will be individually and clearly labeled either ‘intramuscular’ or ‘intranasal’. During the course of the trial only Pharmpackpro Pty Ltd will know which packs contain either active Naloxone preparation or placebo, or intramuscular preparation or placebo.

Pharmpackpro Pty Ltd will provide the research co-ordinator with a code assigned to each treatment arm but that does not reveal whether the arm contains the active Naloxone or placebo. In this way, the two treatment arms will be distinguishable for fortnightly analysis, but the researchers will remain blinded. The packs will be

sequentially numbered, and coded according to treatment arm, and the pack number and code used during the trial will be recorded, along with the other clinical observations by the MSIC staff on the data-form. In this way, the participants, the nurses administering the Naloxone and the researchers will be blinded to the two treatment arms.

Unblinding will occur in the following way: once all participants have been recruited, and the active phase of the trial has concluded, clinically-significant differences in the responses of the participants will be analysed. After this analysis, treatment allocations will be de-coded by obtaining from Pharmpackpro Pty Ltd. the numbers of the packs and codes that correspond to the two treatment arms, thereby revealing to which treatment arm each of the participants belongs.

8 STATISTICAL CONSIDERATIONS

Recently, the study findings of a RCT comparing IN and IM Naloxone have been reported.[13] In that study, requirement for a secondary dose of Naloxone was reported as IN:18% vs IM:4%. We estimate (using JavaStat) that we would need a sample size of 99 per group to achieve adequate power (power=0.8, with 95% confidence) to detect a difference of 14% in requirement for secondary Naloxone.

Descriptive analyses (proportion, median, effect size difference with 95% confidence intervals) will be conducted to compare demographic characteristics and over-dose related variables for the two groups by allocation (IN and IM) (e.g., age, gender demographic data, prior drug use, previous alcohol use, etc).

Primary outcomes will be compared by univariate analysis including odds ratio with 95% CI, hazard ratio (HR) and chi-square analysis. Response time will be compared using a Kaplan-Meier survival curve. A clinically significant difference in response time will be defined as two minutes.

For cases in which response times are not recorded on patient recovery, and subsequent discharge from the treatment room, a default figure of 10 minutes will be recorded.

9 STORAGE AND ARCHIVING OF STUDY DOCUMENTS

Study documents, both hard and soft copies, will be stored in accordance with the Australian Code for the Responsible Conduct of Research. Data-forms will be secured in a locked filing cupboard in a locked office at the MSIC. Only the research team will have access to this filing cabinet.

Data will be entered into an electronic database that will be password protected. Only study investigators will have access to the database.

Data will be kept for a period of 15 years after publication of study findings in a peer-reviewed medical journal. At that time, paper files will be destroyed by paper shredder and electronic files will be deleted from computers.

10REFERENCES

1. Hussain A, Kimura R, & Huang C. Nasal absorption of Naloxone and buprenorphine in rats. Int J Pharm 1984; 21:233.
2. Loimer N, Hofmann P, & Chaudry H. Nasal administration of Naloxone is as effective as the intravenous route in opiate addicts. Int J Addic. 1994 Apr; 29 (6) 819-827.

3. Kerr D, Dietze P, & Kelly AM. Intranasal Naloxone for the treatment of suspected heroin overdose. Review article. Addiction. 2008 Mar: 103(3) 379-86
4. Ashton H, & Hassan Z. Best evidence topic report. Intranasal Naloxone in suspected opioid overdose. Emerg Med J 2006; 23: 221-223
5. Wolfe TR & Bernstone T. Intranasal drug delivery: an alternative to intravenous administration in selected emergency cases. J Emerg Nurs 2004; 30(2): 141-7.
6. Costantino HR, Illum L, Brandt G, Johnson PH & Quay SC. Intranasal delivery: physicochemical and therapeutic aspects. Int J Pharm 2007; 337(1-2): 1-24.
7. Loimer N, Hofmann P & Chaudhry HR. Nasal administration of Naloxone for detection of opiate dependence. J Psychiatr Res 1992; 26(1): 39-43.
8. Barton ED, Colwell CB, Wolfe T, Fosnocht D, Gravitz C, Bryan T., et al. Efficacy of intranasal Naloxone as a needleless alternative for treatment of opioid overdose in the prehospital setting. J Emerg Med 2005; 29(3): 265-71.
9. Barton E.D., Ramos J., Colwell C., Benson J., Baily J., Dunn W. Intranasal administration of Naloxone by paramedics. Prehosp Emerg Care 2002; 6(1): 54-8.
10. Kelly A.M., Koutsogiannis Z. Intranasal Naloxone for life threatening opioid toxicity. Emerg Med J 2002; 19(4): 375.
11. Robertson T., Hendey G., Stroh G., Shalit M. Prehospital intranasal versus intravenous administration of Naloxone for narcotic overdose. Society for Academic Emergency Medicine; 2005; New York; p. 166-7.
12. Kelly A. M., Kerr D., Dietze P., Patrick I., Walker T., Koutsogiannis Z. Randomised trial of intranasal versus intramuscular Naloxone in prehospital treatment for suspected opioid overdose. Med J Aust 2005; 182: 24–7.
13. Kerr, D, Kelly, AM, Dietze, Paul, Jolley, D., Barger, B. Randomized controlled trial comparing the effectiveness and safety of intranasal and intramuscular naloxone for the treatment of suspected heroin overdose. Addiction. 2009: 104, 2067-2074

11APPENDICES

1. Advertisement(s): please see appended PICF

2. Data collection sheet / Case Report Form: please see appended data collection form

Statistical analysis Plan - final

Analyses will be conducted in accordance with the International Conference on Harmonization E9 statistical principles [Lewis, 1999], and reported according to the CONSORT recommendations [Moher et al, 2001; Hopewell et al, 2008]. An intention-to-treat analysis will be performed for all participants who were randomized to receive either mode of treatment (IN and IM administration).

Descriptive analyses will be conducted to compare trial arms (IN and IM) for observed differences in demographics and other important clients' characteristics.

As the trial progressed it was noted that there were several individuals who experienced multiple overdoses who were randomized into the study, introducing clustering. Therefore the main analysis for comparing the incidence rate of secondary naloxone administration mode (IN and IM) will be performed using Generalized Linear Mixed Effects (GLMM) analysis [McCulloch, 2001] for a binary outcome variable with a logistic link function to account for within-client clustering effects because of the multiple overdoses. A Generalized Estimating Equation (GEE) technique with unstructured covariance pattern will be implemented for this purpose. Odds ratio (OR) and 95% confidence interval (CI) will be reported as intervention impact, as per the original analysis plan.

For secondary time to event outcomes medians and 95% CIs at each study arm and Kaplan-Meier survival plots will be reported as descriptive measures. Cox proportional hazards regression models, with shared Gamma frailties to account for within-client clustering effects, will be used to compare between group hazard rates. Hazard ratio (HR) and 95% CI will be reported as intervention impact as per the original analysis plant. We will explore the proportional hazards assumption through visual inspection of survival and hazard plots.

In order to fully explore the effects of repeat presentations, secondary analyses involving only the first overdose of clients will be conducted using analogous methods for primary binary (logistic regression) and secondary time to event (Cox proportional hazards) outcomes. All analyses will be carried out using Stata (version 15; Stata, College Station, TX).

References:

Lewis JA. Statistical principles for clinical trials (ICH E9): an introductory note on an international guideline. Stat Med. 1999;18(15):1903-42.

McCulloch, C.E. and Neuhaus, J.M., 2001. Generalized linear mixed models. John Wiley & Sons, Ltd.

Moher D, Schulz KF, Altman DG (2001) The CONSORT statement: revised recommendations for improving the quality of reports of parallel-group randomised trials. Lancet 357:1191–1194

Hopewell S, Clarke M, Moher D et al (2008) CONSORT Group. CONSORT for reporting randomised trials in journal and conference abstracts. Lancet 371:281–283
