# Supplementary material for: Effect of Intranasal vs Intramuscular Naloxone on Opioid Overdose: A Randomized Clinical Trial
Source: JAMA Netw Open. 2019 Nov 13;2(11):e1914977. doi: 10.1001/jamanetworkopen.2019.14977 (PMC6902775; doi:10.1001/jamanetworkopen.2019.14977)
Supplement: Supplement 2. — eAppendix. Analysis of First Client Presentations eTable. Between Group Analysis of Main Outcome Measures—First Presentations Only [file jamanetwopen-2-e1914977-s002.pdf]

Supplementary Online Content

Dietze P, Jauncey M, Salmon A, et al. Effect of intranasal vs intramuscular naloxone on opioid overdose: a randomized clinical trial. *JAMA Netw Open.* 2019;2(11):e1914977. doi:10.1001/jamanetworkopen.2019.14977

eAppendix. Analysis of First Client Presentations

eTable. Between Group Analysis of Main Outcome Measures—First Presentations Only

This supplementary material has been provided by the authors to give readers additional information about their work.

© 2019 Dietze P et al. *JAMA Network Open*

eAppendix. Analysis of First Client Presentations

Analyses were repeated, but only included the first presentation for each client. (Table 4). There were 60 clients who received intramuscular naloxone at the first presentation, and 67 clients who received intranasal naloxone. Clients randomised to intramuscular naloxone administration were less likely to require secondary naloxone (5.0%), as compared to intranasal naloxone administration (26.9%) (Odds Ratio 0.14; 95% CI: 0.04-0.52). For time to event data, the median time to adequate GCS (e.g., at least 13) was 7.0 minutes (95% CI, 5.3-8.7) with intramuscular administration as compared with 15.0 minutes (95%CI, 13.7-16.3) for intranasal administration (Hazard ratio 1.68, 95% CI 1.14-2.47). The median time to adequate respirations (at least 10 per minute) was 8.0 minutes (95%CI, 5.1-10.9) with intramuscular administration as compared with 16.0 minutes (95%CI, 13.5-18.5) for intranasal administration (Hazard ratio 1.69, 95% CI 1.10-2.58).

© 2019 Dietze P et al. *JAMA Network Open*

eTable. Between Group Analysis of Main Outcome Measures—First Presentations Only

**Outcome**		**Intramuscular**	**Intranasal**	**Intervention Impact (95% CI)**
**Secondary naloxone n (%)**	**Yes**<br>**No**	3 (5.0)<br>57 (95.0)	18 (26.9)<br>49 (73.1)	0.14[1]<br>0.04-0.52
**Time (minutes) to GCS ≥ 13/15**	**Median**<br>**95% CI**<br>**n**	7<br>5.3-8.7<br>55	15<br>13.7-16.3<br>59	1.68[2]<br>1.14-2.47
**Time (minutes) to RR ≥10**	**Median**<br>**95% CI**<br>**n**	8<br>5.1-10.9<br>47	16<br>13.5-18.5<br>61	1.69[2]<br>1.10-2.58

1. Odds Ratio and 95% Confidence Intervals for dichotomised outcomes
2. Hazards Ratio for time to event outcomes.

© 2019 Dietze P et al. *JAMA Network Open*
