# Supplementary material for: Effect of Intranasal vs Intramuscular Naloxone on Opioid Overdose: A Randomized Clinical Trial
Source: JAMA Netw Open. 2019 Nov 13;2(11):e1914977. doi: 10.1001/jamanetworkopen.2019.14977 (PMC6902775; doi:10.1001/jamanetworkopen.2019.14977)
Supplement: Supplement 3. — Data Sharing Statement [file jamanetwopen-2-e1914977-s003.pdf]

Data Sharing Statement

Dietze. Effect of Intranasal vs Intramuscular Naloxone on Opioid Overdose. *JAMA Netw Open*. Published November 13, 2019.
10.1001/jamanetworkopen.2019.14977

Data
Data available: No

Additional Information
Explanation for why data not available: This was not considered a part of the original ethics submission which was made some time ago. We would entertain any request on a case-by-case basis.
